# Influence of iron supplementation on fatigue, mood states and sweating profiles of healthy non-anemic athletes during a training exercise: A double-blind, randomized, placebo-controlled, parallel-group study

**DOI:** 10.1016/j.conctc.2023.101084

**Published:** 2023-02-03

**Authors:** Mahendra P. Kapoor, Masaaki Sugita, Mikiko Kawaguchi, Derek Timm, Aki Kawamura, Aya Abe, Tsutomu Okubo

**Affiliations:** a Taiyo Kagaku Co., Ltd., Research & Development, Nutrition Division, 1-3 Takaramachi, Yokkaichi, Mie, 510-0844, Japan; b Nippon Sport Science University, Faculty of Sport Science, 7-1-1 Fukusawa, Setagaya-Ku, Tokyo, 158 8508, Japan; c Otsuma Women's University, Faculty of Home Economics, Department of Food Science, 12 Sanbancho, Chiyoda, Tokyo, 102-8357, Japan; d Taiyo International Inc, 5960 Golden Hills Dr., Minneapolis, MN, 55416, USA

**Keywords:** Iron supplementation, Non-anemic athletes, Exercise, Sweating, Fatigue, Salivary biomarkers, Profile of mood states (POMS)

## Abstract

Iron is specifically important to athletes, and attention has grown to the association between sports performance and iron regulation in the daily diets of athletes. The study presents new insights into stress, mood states, fatigue, and sweating behavior among the non-anemic athletes with sweating exercise habits who consumed a routine low dose (3.6 mg/day) of iron supplementation. In this double-blind, randomized, placebo-controlled, parallel-group study, both non-anemic male (N = 51) and female (N = 42) athletes were supplemented either with a known highly bioavailable iron formulation (SunActive® Fe) or placebo during the follow-up training exercise period over four weeks at their respective designated clinical sites. The effect of oral iron consumption was examined on fatigue, stress profiles, as well as the quality of life using the profile of mood state (POMS) test or a visual analog scale (VAS) questionnaire, followed by an exercise and well-being related fatigue-sweat. Also, their monotonic association with stress biomarkers (salivary α-amylase, salivary cortisol, and salivary immunoglobulin A) were determined using spearman's rank correlation coefficient test. Repeated measure multivariate analysis of variance (group by time) revealed that the total mood disturbance (TMD) score was significantly lower (P = 0.016; F = 6.26) between placebo and iron supplementation groups over the four weeks study period among female athletes. Also, a significant reduction in tired feeling/exhaustion after the exercise (P = 0.05; F = 4.07) between the placebo and iron intake groups was noticed. A significant within-group reduction (P ≤ 0.05) was noticed in the degree of sweat among both male and female athletes after 2 and 4 weeks of iron supplementation, while athletes of the placebo intake group experienced a non-significant within-group reduction in the degree of sweat*.* Overall, the result indicates routine use of low dose (3.6 mg/day) iron supplementation is beneficial for non-anemic endurance athletes to improve stress, mood states, subjective fatigue, and sweating conditions.

## Highlights

• Sweat iron lose is crucial for non-anemic endurance athletes during exercise sweating.
• Effect of iron consumption was examined on fatigue and stress using POMS test and VAS scale.
• The routine use of low dose (3.6 mg/day) iron supplementation is beneficial for athletes.
• Iron supplementation can help maintain and improve the wellness of non-anemic athletes.

## Graphical abstract

**Figure:**
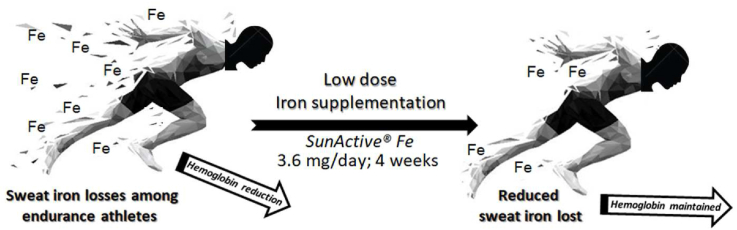

## Teaser

Simple Summary: Iron deficiency is frequent among athletes. Low dietary iron intake can have a negative impact on physical performance and endurance in non-anemic males and more often in females. Also, the increased sweat iron loss is important in iron balance consideration in athletes to prevent a decline in iron status during training. In this study, we investigate the effect of a routine low dose of iron supplementation on the fatigue, mood states, and sweating behavior of both non-anemic male and female athletes. It is recommended that even low-dose iron supplementation (3.6 mg/day) could be helpful to maintain and ameliorate the overall wellness of non-anemic athletes engaged in endurance exercise, as well as individuals with marked sweat losses due to heavy physical work.

## 1 Introduction

Iron is an essential nutrient for the functioning of the various biochemical process including gene regulation, regulation of cell growth, immune function, and neurotransmitter system functions [1]. Iron and other nutritional deficiencies can result in hemoglobin (Hb) abnormalities causing decreased red blood cell production [2]. Also, Iron is required for optimal mitochondrial functions of many oxidative enzymes and proteins regulating the intracellular metabolism [1,[3], [4], [5], [6], [7]].

Iron deficiency is frequent among endurance athletes with excessive training regimens [[8], [9], [10], [11], [12], [13], [14]]. The majority of studies have shown an iron deficiency in athletes from a variety of sporting disciplines [[15], [16], [17], [18]] that require a high level of fitness to sustain proficient, physical, and cognitive functions. A lack of iron can strongly affect physical work capacity by reducing oxygen transport to muscles [1]. Therefore, the recommended iron needs are higher in physically active individuals compared to sedentary individuals. When adequate Hb levels (men ≥13 g/dL, women ≥12 g/dL) are not maintained, poor physical performance and reduced exercise capacity are observed in athletes [[19], [20], [21]].

Furthermore, the loss of iron in sweat is generally higher in athletes undergoing intensive training than in sedentary healthy individuals. Buono et al. reported a significantly higher peripheral sweat rate in trained men and women compared with sedentary men and women [22]. In particular, women have an increased risk of low iron status as a result of exercise-related iron loss combined with iron loss due to menstruation [1,3,11,23,24]. Also, iron insufficiency can have a negative impact on physical performance, and athletes may suffer from non-specific symptoms such as fatigue, weakness, and lethargy [13,25,26]. The mechanisms that cause iron loss during exercise are hemolysis due to mechanical forces and oxidative stress, gastrointestinal and urinary tract bleeding due to microscopic lesions, and extreme sweating, which can result in iron deficiency [13]. Sweat excretion from eccrine sweat glands is primarily a mechanism of thermoregulation but is also a way the body loses iron [9,27]. For instance, the daily sweat-related iron loss is estimated to be 1–2 mg per 2 h of exercise, equivalent to 1% and 3% of recommended daily intake of iron for women and men respectively [18,[28], [29], [30]]. Iron is necessary for oxygen transport and energy metabolism among endurance athletes to maintain their exercise capacity, and to prevent increased heart rate, shortness of breath, and exhaustion during exercise. Therefore, maintaining the iron level in the body is an important step because only a small portion of iron is absorbed in the body from the iron supplementation uptakes.

Several studies have examined the association between iron deficiency and non-specific symptoms particularly fatigue, mood states, and emotional behavior among athletes. In a randomized, controlled trial [31], suggested the intake of iron supplementation can benefit fatigue in non-anemic menstruating women with low ferritin [6]. recommended young female athletes consider the use of low-dose iron supplements under dietary supervision to maintain iron status during training. In another double-blind, randomized, placebo-controlled trial [25], concluded that iron supplementation attenuates fatigue and improves indicators of iron status and emotional fatigue in female officers-in-training. A study examined the sweat iron losses among female recreational cyclists [29] and suggested iron supplementation may offset iron loss during prolonged exercise [9]. measured sweat iron concentration during a four-week exercise training program (both female and male) to determine the changes in iron excretion and suggested that it may be an indicator of iron deficiency observed among active individuals. Also, a number of studies emphasized the importance of iron balance on optimal work capacity for the physical fitness of athletes [32].

Further, the possible interactions between stress, salivary α-amylase, and salivary cortisol levels have recently been acknowledged. Salivary α-amylase, a major enzyme secreted from the salivary glands in response to sympathetic stimuli, is a novel biomarker for psychosocial stress responsiveness and for evaluating the sympathetic nervous system (SNS) activity [[33], [34]]. Whereas, cortisol is a steroid hormone secreted from the adrenal cortex of the hypothalamic-pituitary-adrenal (HPA) axis that is responsible for stressors including physical exertion [[35], [36], [37]]. Increased cortisol levels are proportionally related to high-intensity exercise among athletes since both HPA and SNS activity can be reliably reflected and represents the changes in salivary cortisol and salivary α-amylase concentrations in response to exercise [[38], [39], [40]]. Recently Pearlmutter et al. reported sweat and salivary cortisol response to stress and nutrition factors, wherein the association between dietary choice, the effect of stress on overall mood, and cortisol secretion are compared between non-athlete and athlete individuals of both sexes [41]. Another study evaluated the stress markers (salivary cortisol and salivary α-amylase), mood states, and sleep indicators in high-level swimmers during a major competition [34]. Salivary immunoglobulin A, a major glycoprotein, plays an important role in the local immune defense system against potential pathogens [42]. Salivary immunoglobulin A concentration levels in saliva are stimulated by various stress factors and may be used to reflect changes in immune function during physical activity [43]. Sari-Sarraf et al. studied the salivary immunoglobulin A response to intermittent and continuous exercise among healthy athletes [44]. While Trochimiak et al. reviewed the function of secretory salivary immunoglobulin A as well as changes caused by the exercise of varying intensity and duration [45]. Okada et al. identified the presence of salivary immunoglobulin A in human sweat and sweat glands and measured the concentration of salivary immunoglobulin A in human sweat by enzyme immunoassay [46].

Although there is no evidence that iron supplementation increases athletic performance [47], however, periods of intense training compromise the ability of the body to maintain adequate iron status potentially requiring iron-rich diets or iron supplements. The recommended dietary intake of total iron is 10 mg/day for adult men and 15 mg/day for menstruating women [[48], [49], [50]]. The balance between iron intake and body iron utilization largely depends on the process by which iron stores are depleted. While iron supplementation is reported to be a more practical way to treat iron deficiency in endurance athletes, however, the benefits of iron supplementation in non-anemic athletes are still unclear. Iron supplementation is also recommended for non-anemic endurance athletes because even mild anemia may substantially decrease the capacity for performance of physical exercise [51,52]. Furthermore, an improvement in the fatigue and sweating conditions of non-anemic athletes as a result of iron supplementation has not yet been evaluated. Therefore, the purpose of this double-blind, randomized, placebo-controlled parallel groups study was to determine whether the fatigue, mood states, and sweating profiles of both male and female non-anemic endurance athletes could be maintained by consumption of a daily low dose (3.6 mg/day) iron supplementation with highly bioavailability iron during the training exercise periods.

## 2 Materials and methods

### 2.1 Study design, ethics, and participants

The present study is a double-blind, randomized, parallel, placebo-controlled intervention study that has been carried out at two separate trial sites for non-anemic male and female endurance athletes. The reason for selecting different sites for male and female participants was simply based on the availability of the athletic subjects. However, each trial site followed a set recruitment strategy as per study protocols. Participants were asked to complete a health history form, and to be eligible, the following criteria had to be met: (i) no major illness, (ii) 20 years of age or over, (iii) no history of iron deficiency (hemoglobin:13.5–14.5 g/dL (for male); 11.5–12.5 g/dL (for female), (iv) no previous history of known fatigue-related pathology, (iv) not under any medication/taking any iron supplements, (v) no history of somatic, or sleep disorder, and (vi) reported to be regularly menstruating (female participants only). Exclusion criteria were: recent surgery, inflammation or pain (chronic or acute), smokers and having a high habitual consumption of caffeine (>100 mg/day) and alcohol (>20 g/day); pregnancy, and breastfeeding. Also, the researchers used an orthopedic questionnaire to confirm the athlete's eligibility to participate in the study.

Fifty-one healthy adult male athletes with regular exercise habits such as professional soccer and futsal player (Criacao Shinjuku, Tokyo, Japan) were recruited for this study. Participants were evaluated at Otsuma Women's University, Tokyo, Japan trial site-1 between October 2019 and November 2019 (average temperature: 18 °C; average humidity: 78%) in an intervention study to determine the influence of dietary iron supplements on fatigue, sweating, and lifestyle profiles of these professional male athletes. The study protocol was approved (No. 2019-035-2) by the Otsuma Women's University Life Science Research Ethics Committee. Also, forty-four healthy adult female athletes with sweating habits and hot yoga experience were evaluated at Nippon Sport Science University, Tokyo, Japan trial site-2 between October 2019 and November 2019 (average temperature: 17.3 °C; average humidity: 77.2%). Sweating habits indicate the fitness level of the athletes. The athletes who are very fit sweat more than their less-fit counterparts during a workout because they need to generate more heat to maximize their evaporative cooling capacity. The study was approved (No. 018-H040) by the Ethics Committee at the Nippon Sport Science University, Tokyo, Japan.

The study procedures were conducted according to the guidelines and ethical standards laid out in the Helsinki Declaration. Procedures were verbally explained to the participants and informed consent was signed by all participating athletes. All participants received detailed information on the purpose of the study (including health hazards, privacy protection, and data management). The study compliance was assured, and the fidelity of the informed intervention was checked.

### 2.2 Lifestyle and diets

At the beginning of the study, the participants were provided verbal and written instructions concerning their lifestyles and diets. Participants were asked to maintain their daily lifestyle during the study period. In particular, subjects were asked not to consume alcohol or energy drinks during the study. To ensure that all participants were roughly in energy balance, each participant kept a food diary for the three consecutive days immediately prior to trial tests. To minimize the respondent burden, the three days’ time period for food records was recommended. Instruction for recording food intakes (meat, fish, eggs, vegetables, milk/dairy products beverages, snacks) was rendered, and a simple dietary survey using the questionnaire was collected to understand the diet intake patterns and nutrient intake status during the study period.227.

### 2.3 Supplementation and dosages

Participants were divided into two groups at each trial site and were assigned to order treatment groups according to a randomization plan of equal block size with a 1:1 allocation ratio to receive either iron supplementation or placebo by an independent researcher. The allocation remained concealed to participants, general instructors, statistical analysts, and principal investigators until the end of the trial. The coding of the groups was revealed to researchers only after all measurements were completed and reported. Participants were instructed to consume the treatment once a day, either before or after meals for four consecutive weeks. The treatments were packaged in the sachet (1.5 g as granules) and were identical in appearance, calorie value (5.7 kcal) taste, and raw materials (dextrin, trehalose, and enzymatically degraded guar gum as stabilizer). The dietary iron sachet contains 3.6 mg of ferric pyrophosphate in granules along with 1.37 g carbohydrates and 1.8 mg sodium (Taiyo Kagaku Cookin Supplement Fe; SunActive Fe®), while the placebo was dextrin (carbohydrates: 1.42 g). The specifications of raw materials and nutrient content of iron and placebo granules supplement were confirmed by an independent accredited laboratory. Compliance was assessed at every visit by counting the number of remaining study product sachets returned by the subjects and using a diary system.

### 2.4 Protocol procedure

The 4-week length of the study was based on the time needed to evaluate non-specific symptoms such as fatigue [9]. The training program (futsal/soccer practice) and exercise regimen for participating male athletes were continued for 4 consecutive weeks (site-1). While the female athletes (site-2) were instructed to execute their habitual exercise at least once a week for about 60 min duration with sweating. Featured exercises performed during the 4 weeks study period were mainly hot yoga, dance exercise, martial art exercise, resistance movements, underwater exercise/swimming, and running/walking. Participants of both groups were asked to record their exercise to ensure that all participants remained in identical physical energy balance during the fitness training during the four weeks study period.

**Fig. 1:**
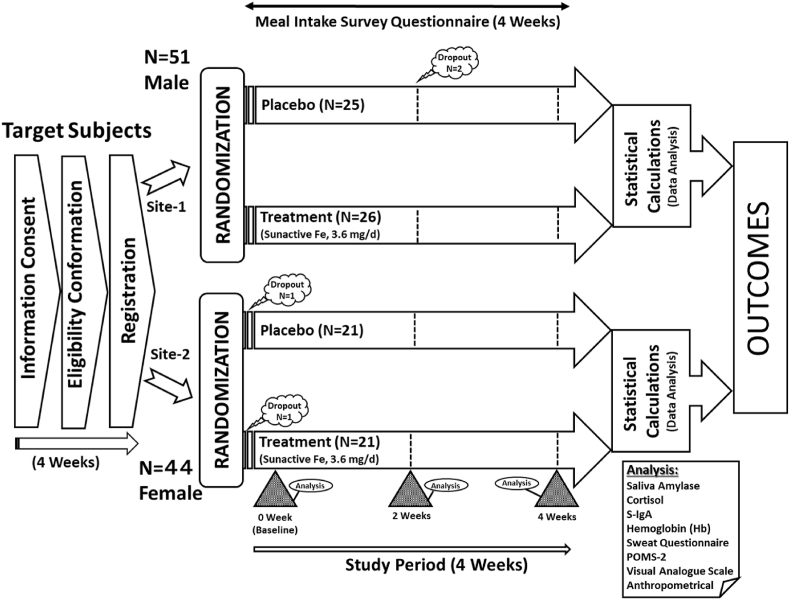
Schematic illustration of experimental design of study protocol and procedures.

A schematic protocol illustration is presented in Fig. 1. Participating male and female athletes were registered for the study at two separate sites after confirmation of their eligibility according to set inclusion/exclusion criteria. All participants were asked to visit their respective testing laboratories at trial sites three times during the four-week study period. During the participant's initial visit (baseline) to the laboratory, a self-completed Profile of Mood State (POMS) questionnaire, and an exercise and well-being related Fatigue-Sweat questionnaire were administrated to male athletes to assess the effect of dietary iron supplementation on quality of life including fatigue and mood symptoms by assessing a range of associated domains. On the other hand, the *state* level of fatigue and related subscale subjective lifestyle quality parameters including sweating behavior among female athletes were measured with a visual analog scale (VAS) questionnaire. The two different questionnaires, VAS and POMS were used for female and male athletic subjects, respectively, because the data collection was based on the required intensities of sweating exercise and metabolism behavior differences among female and male athletes. However, the purpose and questions asked in both questionnaires were interrelated to retain the identical outcomes of the study. Each participant completed the study questionnaires at baseline, after two weeks (mid-point), and after four weeks of the study (end-point). Mood, stress, and sweat behavior were analyzed using the scores obtained from questionnaires.

The anthropometric, body temperature and heart rate were measured for each participant during every laboratory visit. Noninvasive measurement of hemoglobin levels, which measures estimated hemoglobin levels in peripheral blood vessels was performed to scientifically monitor the participating athletes' conditioning on each visit to the laboratory during the study period. Furthermore, to determine the changes in stress markers during the study, saliva samples were collected at the beginning and end of the study, in order, to measure the salivary cortisol, salivary α-amylase, and salivary-immunoglobulin A concentrations.

### 2.5 Study variables assessment

The Profile of Mood States (POMS 2; Second edition; Educational and Industrial Testing Service, San Diego, CA, USA) questionnaire is a series of 65 adjectives (items). POMS-2 describes a self-report assessment of six mood clusters Anger-Hostility (AH), Tension-Anxiety (TA), Depression-Dejection (DD), Fatigue-Inertia (FI), Confusion-Bewilderment (CB), and Vigor-Activity (VA) on a five-point Likert scale from 0 to 4 (0 = not at all; 1 = a little; 2 = moderate; 3 = quite a bit; 4 = extreme), adaptable to capturing fluctuating feelings [[53], [54]]. The total mood disturbance (TMD) score is computed by adding the five negative subscale scores (AH + TA + DD + FI + CB) and subtracting the (VA) score. Friendliness could be considered separately, as a mood cluster that may influence the severity of mood disturbance through interpersonal functioning. The raw scores for each adjective were analyzed to evaluate the changes in mood states. The Japanese version of the POMS-2 (Keneko Shobo Co., Tokyo, Japan) was used in the present study [54] to evaluate the mood states of male athletes.

An additional self-reported Sweat-Fatigue questionnaire was distributed to male athletes to evaluate the athletes conditioning using a rating of perceived exertion scales for sweating and related fatigue conditions (0 = not at all; 1 = a little; 2 = moderate; 3 = quite a bit; 4 = extreme). On the other hand, the *state* level of fatigue among female athletes was measured with a visual analog scale (VAS). The scale comprises a 100-mm line that is anchored with “Negative (*i.e.* fatigue is absent)” on the left and “Positive (*i.e.* most fatigue ever)" on the right. All participating female athletes completed a VAS on various subjective conditions checked at baseline, after two weeks, and four weeks while at rest and post-exercise. The evaluated subjective conditions included fatigue, sleep quality, physical conditions, and related feelings and were reported to determine the effectiveness of iron supplementation among female athletes.

### 2.6 Biomarkers, biochemical analytical methods, and instrumentation

Participants were instructed to rinse out their mouths with bottled water and asked to swallow the remaining saliva before saliva sample collection to eliminate the possibility of any foreign substances that may affect the analytical analysis of salivary cortisol, salivary immunoglobulin-A, and salivary α-amylase. No teeth brushing, flavored or non-flavored chewing gums, and eating/drinking were allowed at least 2 h before the saliva sampling. To allow the spontaneous saliva flow in the mouth participants were seated in a standardized comfortable position throughout the saliva sampling procedure (room temperature, 25 ± 1 °C; relative humidity 52 ± 3%). Participants provided 3–5 mL of saliva samples using technical spitting in a pre-sterilized biological test tube during their initial visit and after completion of the study. The salvia was collected at the same time of the day (between 10:00 to 11:00 a.m.) to avoid the circadian variation in salivary cortisol, immunoglobulin-A, and α-amylase levels. The saliva samples were ice-cooled immediately and stored frozen at −20 °C until analysis. Before the analysis, samples were thawed and saliva samples were prepared by centrifugation at 5000 rpm (2800 xg) for 10 min to isolate any particulate matter and clean samples were used for targeted analytical assays.

The salivary levels of cortisol and immunoglobulin-A were estimated using commercial kits according to the protocols provided by the manufacturers. Each assay was analyzed in duplicate to maintain inter and intra assay coefficient of variations. Salivary cortisol level was analyzed using an ELISA kit assay (limit of detection ≥0.5 ng/mL) according to the method already reported elsewhere [55]. Whereas, salivary immunoglobulin-A concentration level was determined using a sandwich ELISA kit assay following the reported procedure [56]. Wherein, 2% fish gelatin (Sigma) dissolved in TPBS (also used for rinsing) was used for blocking and dilution of saliva samples and HRP-conjugate. The substrate solution, a 10:9:1 mixture of H_2_O_2_ (0.006%) dissolved in a citrate buffer (0.2 M; pH 4.0), H_2_O, and 6 mg/mL of 2, 2′-azino-bis (3-ethylbenzothiazoline-6-sulfonic acid) diammonium salt (Wako Pure Chemicals, Osaka) was used for ELISA. Salivary immunoglobulin A levels were estimated by use of a standard curve. For singlet estimation, the test used 25 μL of saliva sample for cortisol, and 50 μL for salivary immunoglobulin A assay. Data were expressed in nanomoles per liter (nmol/L) and microgram per milliliter (μg/mL), for salivary cortisol and immunoglobulin-A, respectively. Salivary levels of α-amylase were analyzed using a commercially available stress check amylase monitor (Product code 59–014; Nipro Corporation, Japan) as an indicator of the sympathetic nervous system, following the guidelines of the stress values (0–30: no stressed; 31–45: little stressed; 46–60: stressed; 61+: very stressed). A cotton roll was set under each subject's tongue. The cotton roll was then condensed using a syringe and the whole saliva was collected under non-stimulation conditions. The test used the saliva sample collection test strip/chip for singlet α-amylase estimation. Salivary α-amylase data were expressed in kilo-unit per liter (KU/L), wherein, the α-amylase activity that reduced sugars equivalent to 1 μmol/min of maltose was defined as one Unit.

During all three visits to the laboratory, each participant's height was measured using a calibrated portable stadiometer with a sensitivity of 0.1 cm (Seca, Japan), and body weight (mass) was recorded on a digital weighing scale with a sensitivity of 100 g (DP-7200, Daiwa Seihei Co., Ltd., Japan). The body mass index (BMI) was calculated as weight in kilograms divided by height in meters squared. Body temperature and pulse rate were recorded using an electronic thermometer (MC-670) and pulse oximeter (OM35), respectively, manufactured by Omron Corporation, Kyoto, Japan. Due to the high frequencies of hemoglobin variation among athletes, it is necessary to make hemoglobin assessment a daily clinical activity. Noninvasive measurement of estimated hemoglobin concentration level (mg/dL) was performed using an automated ASTRIM FIT Health Monitoring Analyzer (Sysmex Corporation, Japan), which measures estimated hemoglobin levels in peripheral blood vessels without the need for blood sampling using a near-infrared spectroscopic imaging method [57].

### 2.7 Statistical analysis and data processing

The statistical analysis involves a comparison between profiles of repeated measurements obtained during the study in the dietary iron treatment and placebo groups of both female and male athletes at their respective trial sites. The results (scores) of the POMS, VAS, and fatigue-sweat questionnaires, and hemoglobin levels recorded for different items at baseline, after two weeks (mid-point) and four weeks (end-point) of the study were examined using a pre/post cofactor analysis of variance (ANCOVA) and multivariate analysis of covariance (MANCOVA; group-by-time interaction) with repeated measures model. To examine the relationship between salivary cortisol, salivary immunoglobulin A, and salivary α-amylase biomarker concentrations recorded before (at baseline), and after completion of four weeks (end-point) of intervention of either dietary iron supplement or placebo in participating athlete groups, the pre/post ANCOVA model was performed. Anthropometric values were compared using the non-parametric Mann-Whitney U test for independent groups. The comparisons of the measurements (variables) were made using appropriate parametric tests, with the assumption that the parametric and non-parametric analyses appeared to give equivalent result interpretations. All values are expressed as mean ± sem unless otherwise stated. The p-value of ≤0.05 was considered statistically significant, and ≤0.10 was regarded as trending toward significant in terms of clinical relevance [58]. For these analyses, effect size estimates were calculated using Cohen's d estimates where appropriate to examine the clinical significance and clinical relevance of the observed changes. The statistical analyses were performed using the JMP statistical package (software ver. 14 of SAS institute). The spearman's rank correlation coefficient was used to test the monotonic association between the involved continuous variables. The hypothesis tested and confidence intervals were used to address the statistical significance of the results and to estimate the strength of the relationship coefficients within sampled data variables in the athletes. The significance level was set at 0.05 for the correlation analyses (P ≤ 0.5). The study compliance was assured, and the fidelity of the inform intervention was assessed.

## 3 Results

### 3.1 Subject characteristics and quantification of dietary intakes

Demographic information and stress-related biomarkers analysis for study participants at baseline is summarized in Table 1. The average age was 28.2 ± 0.8 and 41.8 ± 1.5 years of male and female athletes, respectively. The average hemoglobin levels of male athletes (14.51 ± 0.19) were slightly higher compared to female athletes (12.59 ± 0.18) and confirming the recruited athletes were non-anemic and healthy individuals. No significant difference (P ≤ 0.05) in age, height, body weight, and body mass index including other parameters were observed at baseline between placebo and Fe treatment groups of either of the designated research sites confirming a homogenous representation of the study subjects. Also, no significant difference in the analysis of hemoglobin, salivary amylase, salivary cortisol, and salivary immunoglobulin-A biomarkers, were observed at baseline among the groups at either clinical site.

**Table 1:** Baseline profiles of the non-anemic athletes were recruited at either clinical site to evaluate the effect of low-dose iron supplementation during four weeks of the training exercise.

Characteristics	Site-1; Male						
	All Subjects (N = 51)		Placebo (N = 25)		Fe-treatment (N = 26)		P- Value^‡^
	*Mean* ± *sem*	*95% CI*	*Mean* ± *sem*	*95% CI*	*Mean* ± *sem*	*95% CI*	(Between groups)
Age (Years)	28.2 ± 0.8	26.7–29.7	28.3 ± 1.1	26.2–30.4	28.2 ± 1.1	26.0–30.3	0.94
Height (Cm)	173.7 ± 0.9	172.0–175.4	173.6 ± 1.2	171.2–176.0	173.8 ± 1.2	171.5–176.2	0.91
Body weight (Kg)	68.6 ± 1.03	66.5–70.6	68.8 ± 1.27	66.3–71.2	68.4 ± 1.62	65.2–71.5	0.85
BMI (Kg/m^2^)	22.7 ± 0.25	22.2–23.2	22.8 ± 0.31	22.2–23.4	22.6 ± 0.39	21.8–23.3	0.65
Hemoglobin (g/dL)	14.51 ± 0.19	14.14–14.88	14.61 ± 0.26	14.11–15.11	14.42 ± 0.29	13.85–14.99	0.63
Amylase (U/L)	15.65 ± 1.80	12.12–19.18	13.04 ± 1.85	9.42–16.66	18.15 ± 3.00	12.26–24.05	0.16
Characteristics	Site-2; Female						
	All Subjects (N = 42)		Placebo (N = 21)		Fe-treatment (N = 21)		P- Value
	Mean ± sem	95% CI	Mean ± sem	95% CI	Mean ± sem	95% CI	(Between groups)
Age (Years)	41.8 ± 1.5	38.9–44.8	41.4 ± 2.0	37.6–45.3	42.2 ± 2.2	37.9–46.6	0.76
Height (Cm)	159.3 ± 0.88	157.6–161.0	159.0 ± 1.2	156.7–161.3	159.6 ± 1.3	157.1–162.2	0.77
Body Weight (Kg)	55.6 ± 1.5	52.6–58.6	55.1 ± 2.0	51.1–59.1	56.1 ± 2.3	51.7–60.6	0.80
BMI (Kg/m^2^)	21.86 ± 0.48	20.92–22.80	21.75 ± 0.63	20.52–22.98	21.96 ± 0.72	20.54–23.38	0.84
Body Temp. (°C)	36.3 ± 0.1	36.2–36.5	36.2 ± 0.1	36.0–36.5	36.4 ± 0.1	36.2–36.6	0.27
Hemoglobin (g/dL)	12.59 ± 0.18	12.24–12.93	12.75 ± 0.24	12.28–13.23	12.42 ± 0.25	11.93–12.91	0.19
Cortisol^#^ (nM)	2.64 ± 0.37	1.91–3.37	3.00 ± 0.69	1.65–4.35	2.28 ± 0.28	1.73–2.82	0.38
s-IgA^#^ (ng/mL)	161.9 ± 16.3	129.9–193.8	179.9 ± 30.6	119.9–239.8	143.8 ± 11.1	122.1–165.6	0.23
Fatigue Condition	0.46 ± 0.04	0.38–0.54	0.49 ± 0.06	0.37–0.61	0.44 ± 0.06	0.34–0.54	0.41
Pulse Rate (bpm)	70 ± 2	67–73	69 ± 2	65–73	70 ± 2	65–74	0.95

#: *N* = *40 (P* = *20, Fe* = *20); bpm* = *beats per minute; N* = *Number of subjects; sem* = *Standard error of mean; CI* = *Confidential interval; Significant P* ≤ *0.05; ‡ Mann-Whitney rank test.*

Of the forty-four accepted female subjects (n = 44) at clinical site-2, who provided informed consent, one subject in each group dropped out of the study due to personal reasons after the randomization. While, of the fifty-one eligible male subjects (n = 51) at clinical site-1 randomized to treatment groups, two subjects of the placebo group did not complete the study, and were dropped out after 2 weeks as they could not wholly comply with the study protocols. Only data collected from the athletes who completed the study were included in the data analysis (Fig. 1). Also, based on the use of a two-sample comparison of means at the alpha = 0.05 level of significance, a total sample size of completers (both female and male subjects) provided 90% power to detect an appropriate effect size.

All athletes were instructed to maintain consistent dietary intakes over the 4 weeks of the study duration and this was confirmed by no significant differences (P ≤ 0.05) were noticed in both male and female athlete's daily dietary habits. The daily dietary consumption among female athletes was lower than male athletes. Further, the breakdown into weekly dietary intake subgroups of all athletes is displayed in Supplementary Table S1. The female athletes were instructed to execute their habitual exercise at least once a week for about 60 min duration with sweating, whereas the average number of days of exercise per week was 2.8 days and 2.9 days in the placebo and Fe-treatment intake groups, respectively, confirming the no significant difference among the groups. Further, the detailed breakdown into weekly exercise regimen subgroups (mostly the hot yoga: temperature 35–39 °C; humidity 60%) of the female athletes is displayed in Supplementary Table S2. The results revealed that athletes in both groups followed a similar exercise regimen over 4 weeks of the study duration.

### 3.2 Profiles of mood state (POMS) assessment

**Fig. 2:**
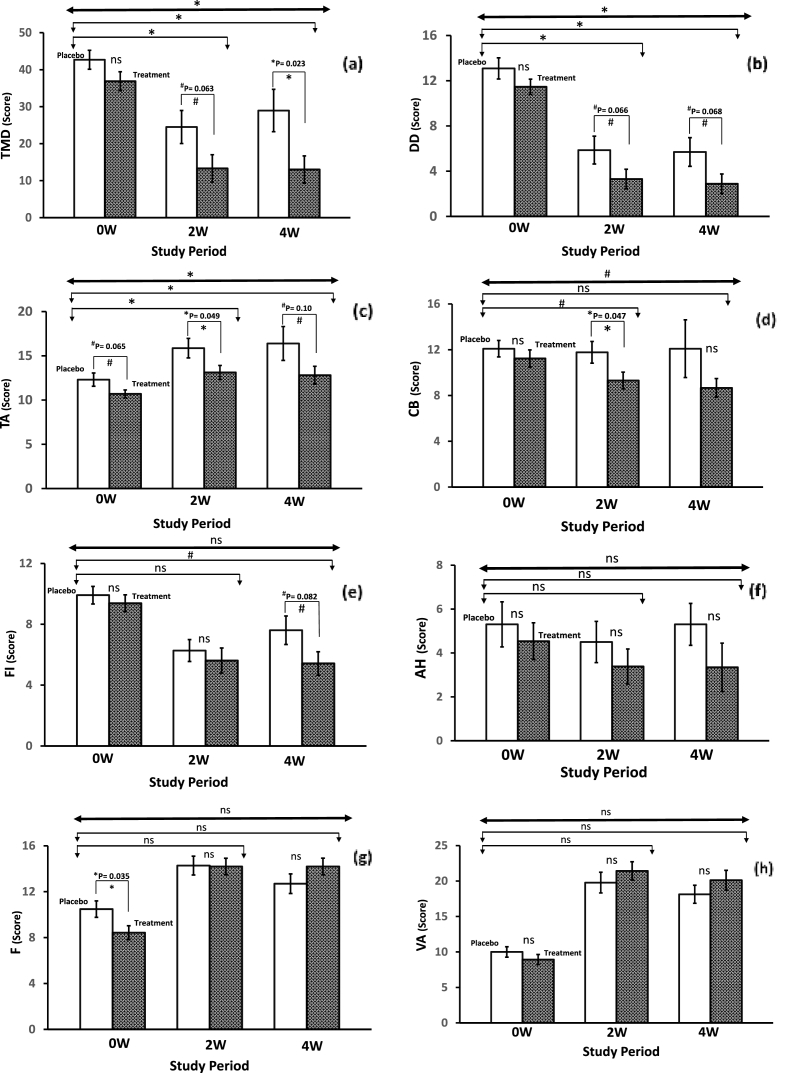
Changes in the profile of mood state (POMS) scores among non-anemic male athletes (professional soccer and futsal players) after either placebo or iron supplementation over four weeks of exercise training. Keys: Hatched bars represent 2x standard error of means (SEM) ranges; NS: Non-significant; *: Significant at P ≤ *0.05; #: Trending significant at P* ≤ *0.10. Line (Dark): Repeated measure MANCOVA; Line (light) with bent arrows: ANCOVA analysis after 2 and 4 weeks of study duration.*

The POMS results summarized in Table 2 indicate the extent of changes in the mood state profiles of the male subjects during the study. The interpretive information is based on an algorithm that reveals the most common interpretation of the obtained scores of mood clusters. There were some significant differences between groups in the POMS scores (95% confidential interval). All negative mood state scales (AH, CB, DD, FI, TA) were found to decrease in the Fe-treatment group when compared to the placebo group (see Fig. 2). Also, the total mood disturbance (TMD) score is determined by summing the above-mentioned negative mood state clusters and subtracting VA (a positive mood state cluster) was lowered in the Fe-treatment group compared to the placebo group, which indicates the extent to which athletes experienced overall negative effects. A non-parametric Mann-Whitney U Rank test showed trending significance when compared after 2 weeks (P = 0.063), and a significant difference after 4 weeks (P = 0.023) among the respective groups (Fig. 2a). Further, the Fe-treatment group showed a significantly lower total mood disturbance after 2 weeks (P = 0.042; F = 4.40), and 4 weeks (P = 0.016; F = 6.31) compared to placebo using an ANCOVA analysis model. Whereas, a repeated measure MANCOVA analysis model (group by time interaction) revealed a significant difference (P = 0.014; F = 6.35) between placebo and Fe-treatment intake during the study period (see Table 2). Further, analysis of individual mood scale scores is defined to identify areas that contribute to the TMD score. Trending significant differences observed for Depression-Dejection (Fig. 2b) and Tension-Anxiety (Fig. 2c) between the placebo and Fe treatment groups in the POMS were revealed significant after 2 and 4 weeks when using an ANCOVA analysis model.

**Table 2:** The changes in the profiles of mood state (POMS) clusters among the non-anemic male athletes during the iron supplementation over four weeks of training exercise regimen compared to placebo (Site 1).

Variables ^σ^	Placebo (N = 23)			*Cohen's d*	Fe-treatment (N = 26)			*Cohen's d*	P-Value & F-Value (*Between groups)*	Repeated measure MANCOVA
	*0W*	*2W*^*#*^	*4W*		*0W*	*2W*	*4W*		*ANCOVA*	(Group by Time)
**Anger-Hostility (AH)**									0W–2W: P = 0.439; F = 0.61	
*Mean* ± *sem*	5.3 ± 1.03	4.5 ± 0.94	5.3 ± 0.95	2W: -0.15	4.5 ± 0.84	3.4 ± 0.80	3.4 ± 1.10	2W: -0.23	*0W–4W: P* = *0.257; F* = *1.32*	P = 0.289; F = 1.11
*95% CI*	3.3–7.3	2.7–6.4	3.4–7.2	4W: 0.01	2.9–6.2	1.8–5.0	1.2–5.5	4W: -0.19		
**Confusion-Bewilderment (CB)**									0W–2W: P = 0.096; F = 2.89	
*Mean* ± *sem*	12.1 ± 0.72	11.8 ± 0.94	12.1 ± 2.52	2W: -0.06	11.2 ± 0.75	9.3 ± 0.74	8.7 ± 0.82	2W: -0.42	*0W–4W: P* = *0.149; F* = *2.15*	P = 0.071; F = 3.37
*95% CI*	10.7–13.5	9.9–13.6	7.1–17.0	4W: 0.00	9.8–12.7	7.9–10.8	7.0–10.3	4W: -0.52		
**Depression-Dejection (DD)**									0W–2W: P = **0.040**; F = 4.49	
*Mean* ± *sem*	13.1 ± 0.94	5.9 ± 1.23	5.7 ± 1.27	2W: -1.10	11.5 ± 0.67	3.3 ± 0.86	2.9 ± 0.86	2W: -1.63	*0W–4W: P* = ***0.041****; F* = *4.41*	P = **0.032**; F = 4.88
*95% CI*	11.3–14.9	3.5–8.3	3.2–8.2	4W: -1.08	10.2–12.8	1.6–5.0	1.2–4.6	4W: -1.71		
**Fatigue-Inertia (FI)**									0W–2W: P = 0.455; F = 0.57	
*Mean* ± *sem*	9.9 ± 0.58	6.3 ± 0.72	7.6 ± 0.94	2W: -0.93	9.4 ± 0.55	5.6 ± 0.83	5.4 ± 0.77	2W: -0.81	*0W–4W: P* = *0.075; F* = *3.32*	P = 0.136; F = 2.35
*95% CI*	8.8–11.1	4.9–7.8	5.8–9.5	4W: -0.47	8.3–10.5	4.0–7.2	3.9–6.9	4W: -0.90		
**Tension-Anxiety (TA)**									0W–2W: P = **0.031**; F = 4.98	
*Mean* ± *sem*	12.3 ± 0.75	15.9 ± 1.10	16.4 ± 1.91	2W: 0.62	10.7 ± 0.45	13.1 ± 0.80	12.8 ± 1.01	2W: 0.55	*0W–4W: P* = ***0.050****; F* = *3.97*	P = **0.028**; F = 5.18
*95% CI*	10.8–13.8	13.7–18.0	12.6–20.1	4W: -0.43	9.8–11.6	11.6–14.7	10.8–14.8	4W: 0.39		
**Vigor-Activity (VA)**									0W–2W: P = 0.762; F = 0.093	
*Mean* ± *sem*	10.0 ± 0.73	19.8 ± 1.46	18.1 ± 1.27	2W: 1.34	8.9 ± 0.72	21.4 ± 1.29	20.1 ± 1.39	2W: 1.77	*0W–4W: P* = *0.686; F* = *0.17*	P = 0.439; F = 0.03
*95% CI*	8.6–11.4	16.9–22.6	15.6–20.6	4W: 1.23	7.5–10.3	18.9–24.0	17.4–22.8	4W: 1.48		
**Total Mood Disturbance (TMD)**									0W–2W: P = **0.042**; F = 4.40	
*Mean* ± *sem*	42.7 ± 2.56	24.5 ± 4.47	29.0 ± 5.72	2W: -0.80	36.9 ± 2.57	13.3 ± 3.70	13.0 ± 3.69	2W: -1.12	*0W–4W: P* = ***0.016****; F* = *6.31*	P = **0.014**; F = 6.35
*95% CI*	37.7–47.7	15.7–33.3	17.8–40.2	4W: -0.48	31.8–41.9	6.1–20.6	5.8–20.2	4W: -1.14		
**Friendliness (F)**									0W–2W: P = 0.196; F = 1.72	
*Mean* ± *sem*	10.5 ± 0.72	14.3 ± 0.82	12.7 ± 0.85	2W:0.83	8.4 ± 0.61	14.2 ± 0.72	14.2 ± 0.73	2W:1.35	*0W–4W: P* = *0.728; F* = *0.12*	P = 0.745; F = 0.13
*95% CI*	9.1–11.9	12.7–15.9	11.0–14.4	4W: 0.47	7.2–9.6	12.8–15.6	12.8–15.6	4W: 1.34		

*#: N* = *22; N* = *Number of subjects; sem* = *Standard error of mean; CI* = *Confidential interval; ANCOVA* = *Analysis of covariance; MANCOVA* = *Multivariate analysis of covariance (Repeated measure); Significant P* ≤ *0.05; Trending significant P* ≤ *0.10;σ*: *Five-point Likert scale from 0 to 4 (0 = not at all; 1 = a little; 2 = moderate; 3 = quite a bit; 4 = extreme); Effect size (Cohen's d)*.

While a repeated measure MANCOVA analysis model also showed the significant differences for Depression-Dejection and Tension-Anxiety mood clusters between the placebo and Fe-treatment groups during the study period. There was significance after 2 weeks for Confusion-Bewilderment, but the trending significance was observed when the pre/post ANCOVA model was applied (Fig. 2d). Although there was no significant difference in the scores after 4 weeks; however, there was a trending significant difference for the Confusion-Bewilderment mood cluster between the placebo and Fe-treatment groups (repeated measure MANCOVA analysis). There was only trending significance for Fatigue-Inertia after 4 weeks comparing the placebo and Fe-treatment (Fig. 2e), while no significant differences for Anger-Hostility were observed (Fig. 2f). Further, no significant differences were noted for Friendliness (Fig. 2g) or Vigor-Activity (Fig. 2h) clusters score between the Fe-treatment and placebo group (see Table 2). Indication of the magnitude of treatment effect is provided by effect size estimates, using Cohen's d, based on the baseline (0W) to follow-up changes in group means after two- and four-weeks of the intervention. The effect sizes associated with iron supplementation on the statistically defined POMS variables were consistently higher compared to placebo (Table 2).

### 3.3 Assessment of exercise-driven study variables

**Fig. 3:**
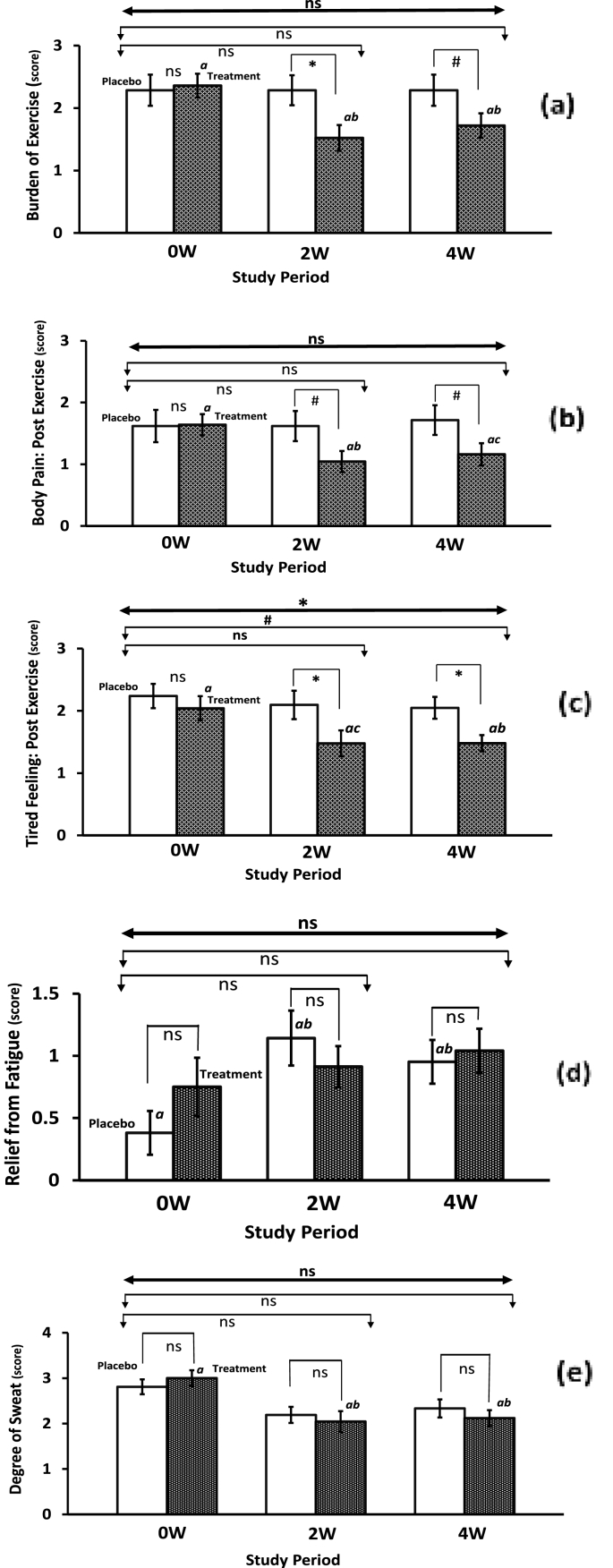
Impact of low dose iron supplementation on the fatigue, sweating, and conditioning of non-anemic male athletes during the four weeks exercise regimen. ***Keys:****As illustrated in*Fig. 1*. Mean bars followed by the different letters indicate a within-group statistical difference. Mean bars followed by the same letter or without any letter indicate a lack of within-group significant difference.*

The results of exercise-related variables (stress, fatigue, and sweat behavior) assessed by *visual analog scale* (VAS) and fatigue-sweat questionnaires for all eligible athletes are summarized in Table 3 and Fig. 3. The male athletes of Fe-treatment group experienced a within-group reduction in the burden of exercise after 2 and 4 weeks, whereas a significant difference after 2 weeks (P ≤ 0.05), and a trending significant difference after 4 weeks (P ≤ 0.10) were noticed when compared to the placebo group (Fig. 3a). However, between groups, statistical significance was not achieved when ANCOVA and repeated measured MANCOVA model was applied. In the iron intake group, 12% of athletes reported no exercise burden compared to none at baseline, whereas in the placebo group only none of the athletes reported no post-exercise burden compared to 4.8% at baseline. The results confirm the amelioration in the athlete's physical condition after iron supplementation over the 4 week study period. Also, a within-group significant reduction was noticed in the post-exercise body pain among the male athletes of the Fe-treatment intake group after 2 weeks (P ≤ 0.05) along with a trending significant reduction after 4 weeks (P ≤ 0.10), while athletes of the placebo intake group reported an elevated post-exercise body pain symptoms. Whereas, a trending significant difference (P ≤ 0.10) after 2 and 4 weeks compared to the placebo group was observed (Fig. 3b), however, no significance between groups was found when ANCOVA and repeated measured MANCOVA model was applied (See Table 3). In the iron intake group, 24% of athletes reported no post-exercise body pain symptoms compared to 4% at baseline, whereas in the placebo group only 9.5% of athletes reported no post-exercise body pain symptoms compared to 14.5% at baseline, further confirming improvements in the athletes' physical condition after iron supplementation over the 4 week study period.

**Table 3:** Differences in the exercise-related variables related to fatigue, sweating, and conditioning among the non-anemic athletes during the iron supplementation compared to placebo over four weeks of the training exercise.

Male (site-1) Variables ^λ^	Placebo (N = 21)			*Cohen's d*	Fe-treatment (N = 25)			*Cohen's d*	P-Value & F-Value (*Between groups)*	Repeated measure MANCOVA
	*0W*	*2W*	*4W*		*0W*	*2W*^*#*^	*4W*		*ANCOVA*	(Group by Time)
**Burden of Exercise**									0W–2W: P = 0.352; F = 0.89	
*Mean* ± *sem*	2.29 ± 0.25	2.29 ± 0.24	2.29 ± 0.25	2W: 0.00	2.36 ± 0.19	1.52 ± 0.21	1.72 ± 0.20	2W: -0.70	*0W–4W: P* = *0.246; F* = *1.39*	P = 0.147; F = 2.26
*95% CI*	1.80–2.78	1.81–2.76	1.80–2.78	4W: 0.00	1.99–2.73	1.12–1.93	1.34–2.10	4W: -0.54		
**Body Pain: Post Exercise**									0W–2W: P = 0.237; F = 1.44	
*Mean* ± *sem*	1.62 ± 0.26	1.62 ± 0.24	1.71 ± 0.24	2W: 0.00	1.64 ± 0.17	1.04 ± 0.17	1.16 ± 0.18	2W: -0.58	*0W–4W: P* = *0.302; F* = *1.09*	P = 0.121; F = 2.53
*95% CI*	1.10–2.13	1.14–2.10	1.24–2.19	4W: 0.07	1.30–1.98	0.71–1.38	0.81–1.51	4W: -0.44		
**Tired Feeling: Post Exercise**									0W–2W: P = 0.132; F = 2.37	
*Mean* ± *sem*	2.24 ± 0.19	2.10 ± 0.23	2.05 ± 0.18	2W: -0.12	2.04 ± 0.20	1.48 ± 0.21	1.48 ± 0.13	2W: -0.46	*0W–4W: P* = *0.075; F* = *3.33*	P = **0.047**; F = 4.27
*95% CI*	1.86–2.62	1.65–2.54	1.70–2.39	4W: -0.19	1.66–2.42	1.07–1.88	1.22–1.74	4W: -0.59		
**Relief from Fatigue**									0W–2W: P = 0.484; F = 0.50	
*Mean* ± *sem*	0.38 ± 0.18	1.14 ± 0.22	0.95 ± 0.18	2W: 0.66	0.75 ± 0.24^φ^	0.91 ± 0.17	1.04 ± 0.18	2W: 0.14	*0W–4W: P* = *0.238 F* = *1.43*	P = 0.356; F = 0.89
*95% CI*	0.04–0.73	0.71–1.58	0.61–1.30	4W: 0.58	0.29–1.21	0.59–1.24	0.69–1.39	4W: 0.24		
**Degree of Sweat**									0W–2W: P = 0.911; F = 0.01	
*Mean* ± *sem*	2.81 ± 0.16	2.19 ± 0.18	2.33 ± 0.20	2W: -0.64	3.00 ± 0.17	2.04 ± 0.23	2.12 ± 0.18	2W: -0.76	*0W–4W: P* = *0.958; F* = *0.03*	P = 0.954; F = 0.02
*95% CI*	2.49–3.13	1.84–2.54	1.94–2.72	4W: -0.45	2.66–3.34	1.59–2.50	1.77–2.47	4W: -0.82		

Female (site-2) Variable^δ^	Placebo (N = 21)				Fe-treatment (N = 25)				P-Value & F-Value (*Between groups)*	Repeated measure MANCOVA
	*0W*	*2W*	*4W*		*0W*	*2W*	*4W*		*ANCOVA*	(Group by Time)
**Burden of Exercise**									0W–2W: P = 0.753; F = 0.10	
*Mean* ± *sem*	52.8 ± 3.59	50.5 ± 5.23	50.9 ± 5.18	2W: -0.09	54.8 ± 4.90	53.3 ± 4.3	48.4 ± 5.5	2W: -0.06	*0W–4W: P* = *0.657; F* = *0.20*	P = 0.789; F = 0.29
*95% CI*	45.8–59.8	40.2–60.8	40.7–61.1	4W: -0.07	45.2–64.4	44.8–61.8	37.7–59.2	4W: -0.22		
**Body Pain: Post Exercise**									0W–2W: P = 0.398; F = 0.73	
*Mean* ± *sem*	55.1 ± 5.76	55.0 ± 5.92	64.9 ± 5.30	2W: 0.00	67.2 ± 5.99	66.0 ± 5.12	61.7 ± 5.47	2W: -0.04	*0W–4W: P* = *0.349; F* = *0.90*	P = 0.152; F = 1.98
*95% CI*	43.8–66.4	43.4–66.6	54.5–75.3	4W: 0.32	55.5–79.0	56.0–76.1	51.0–72.4	4W: -0.17		
**Tired Feeling: Post Exercise**									0W–2W: P = 1.0; F = 0.0	
*Mean* ± *sem*	46.0 ± 3.71	53.8 ± 5.34	54.5 ± 4.26	2W: 0.28	44.6 ± 4.04	52.9 ± 4.87	56.9 ± 4.45	2W: 0.32	*0W–4W: P* = *0.673; F* = *0.18*	P = 0.848; F = 0.19
*95% CI*	38.7–53.3	43.3–64.2	46.2–62.9	4W: 0.37	36.7–52.5	43.3–62.4	48.1–65.6	4W: 0.51		
**Relief from Fatigue**									0W–2W: P = 0.264; F = 1.28	
*Mean* ± *sem*	56.9 ± 3.21	64.0 ± 3.83	67.0 ± 3.68	2W: 0.35	63.2 ± 4.14	59.9 ± 3.31	63.9 ± 4.38	2W:- 0.17	*0W–4W: P* = *0.323; F* = *1.00*	P = 0.126; F = 2.27
*95% CI*	50.6–63.2	56.5–71.5	59.8–74.2	4W: 0.51	55.1–71.4	53.4–66.3	55.4–72.5	4W: 0.03		
**Degree of Sweat**									0W–2W: P = **0.054**; F = 3.94	
*Mean* ± *sem*	72.6 ± 5.54	76.5 ± 3.62	69.8 ± 4.18	2W: 0.16	77.1 ± 3.26	63.2 ± 5.75	66.6 ± 4.57	2W: -0.49	*0W–4W: P* = *0.606; F* = *0.27*	P = 0.079; F = 2.74
*95% CI*	61.8–83.5	69.4–83.6	61.6–77.9	4W: -0.11	70.7–83.5	51.9–74.5	57.7–75.6	4W: -0.45		

*#: N* = *23;* φ*: N* = *24; N* = *Number of subjects; sem* = *Standard error of mean; CI* = *Confidential interval; ANCOVA* = *Analysis of covariance; MANCOVA* = *Multivariate analysis of covariance (Repeated measure); Significant P* ≤ *0.05; Trending significant P* ≤ *0.10;* λ: *Rating of perceived exertion, sweating and fatigue (0 = not at all; 1 = a little; 2 = moderate; 3 = quite a bit; 4 = extreme).* δ*: Visual analog scale (VAS); Effect size (Cohen's d)*.

A within-group trend in reduction of tired feeling after the exercise was observed in the Fe-treatment group after 2 weeks and 4 weeks. Likewise, a clear significant difference (P ≤ 0.05) was noticed after 2 and 4 weeks of iron supplementation compared to the placebo group for tired feeling (Fig. 3c). Whereas a trending significance (P = 0.075; F = 3.33) was noted after 4 weeks of Fe-treatment intake compared to placebo when using a pre-post ANCOVA model, and the repeated measure MANCOVA analysis model further confirmed a significant difference (P = 0.047; F = 4.27) for the tired feeling after the exercise between the placebo and iron intake groups over the study period. Although a within-group gradual increase in the relief from fatigue was noticed for both placebo and Fe-treatment groups, no significant levels were observed between groups (Fig. 3d). Further, a within-group significant reduction (P ≤ 0.05) was noticed in the degree of sweat among the athletes of the Fe-treatment group after 2 and 4 weeks, while athletes of the placebo group experienced a non-significant reduction in the degree of sweat (Fig. 3e)*.* No significant differences in sweating were observed when compared to the placebo group (see Table 3). Noteworthy to mention that in the iron intake group, the relief from the fatigue was also effectively reduced (i.e. very fatigue 36% and less fatigue 60% at baseline to very fatigue 4% and less fatigue 92% after iron supplementation over 4 weeks), while no difference in the placebo group was noticed*.*

**Fig. 4:**
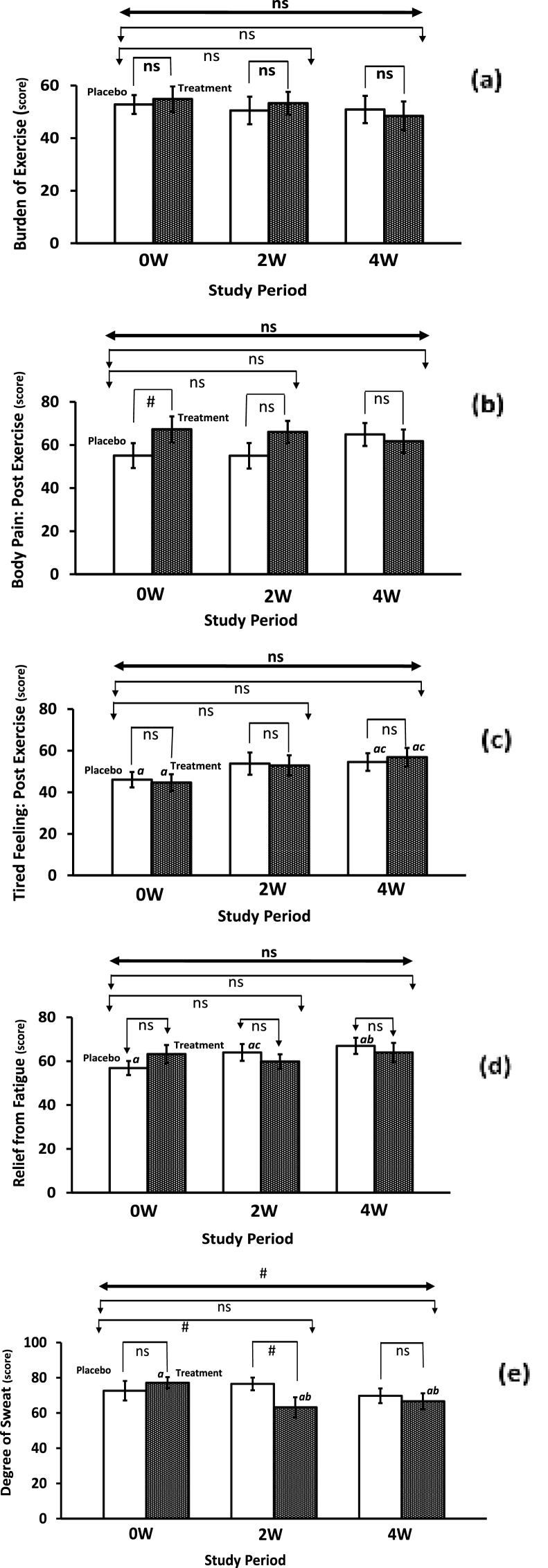
Influence of low dose iron supplementation on the fatigue, sweating, and rendered physical profiles of non-anemic female athletes (experienced hot yoga practitioners) during four weeks training exercise regimen. *Keys: As illustrated in*Fig. 1, Fig. 2*.*

On the other hand, the female athletes in both groups reported no significant differences in the burden of exercise (Fig. 4a), post-exercise body pain (Fig. 4b), tired feeling after exercise (Fig. 4c)*,* and experienced no considerable relief from fatigue (Fig. 4d). However, a within-group significant reduction (P ≤ 0.05) in the degree of sweat was noticed among the female athletes of the Fe-treatment group after 2 and 4 weeks of consumption (Fig. 4e). Also, a trending significant reduction (P = 0.10) was observed only after 2 weeks when compared to the placebo group and was further confirmed to a nearly significant level (P = 0.054; F = 3.94) using the ANCOVA model. In addition, a trending significant level (P = 0.085; F = 2.54) was achieved between placebo and Fe-treatment groups during the study period when a repeated measured MANCOVA model was applied (See Table 3). Again, after two- and four weeks, group differences in above mentioned self-report scores of variables were in the direction favoring iron supplementation were generally not statistically significant, while few trended toward significant (P < 0.10), and in the small to medium range of effect size, as indexed by Cohen's *d* in Table 3.

Considering the sleep efficacies evaluation, the athletes of the Fe-treatment intake group reported better sleep quality as well as less sleepiness. Overall, the total sleep efficiency score was improved over the 4-week study period when compared to the placebo group (Supplementary Table S3), which also confirmed the amelioration of fatigue among the athletes. No significant differences in pulse rate and body temperature could be noticed between placebo and Fe-treatment groups (P > 0.05), while a significant difference in the body weight (P = 0.006; F = 2.53) was observed between groups among the female athletes over the four-week study duration (Supplementary Table S3).

### 3.4 Biological variables quantification

**Fig. 5:**
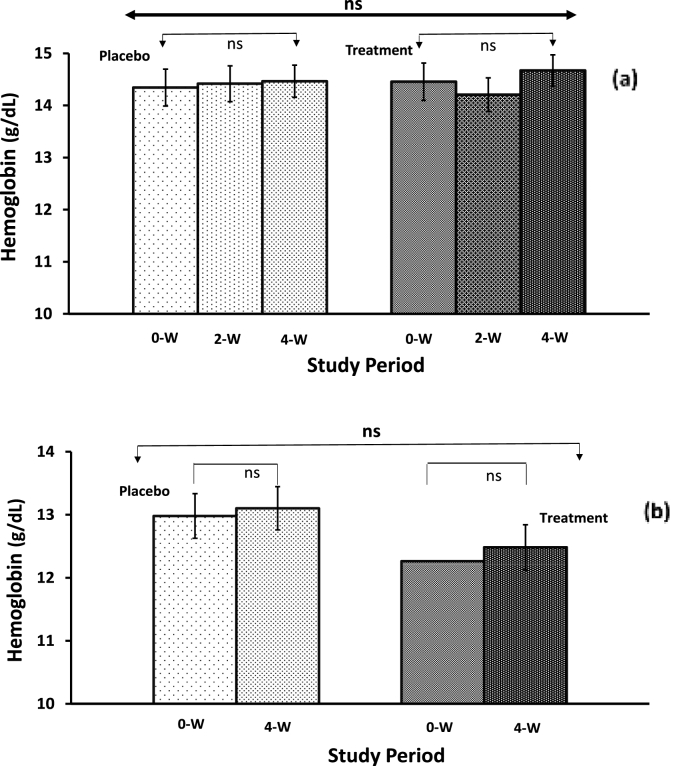
A low dose of iron supplementation helps maintain the hemoglobin levels among non-anemic athletes during the routine exercise training over 4 weeks of the study duration. (a) Male athletes, and (b) female athletes (No significant difference was observed within-groups and between the placebo and iron supplementation groups).

The hemoglobin content and salivary stress biomarkers quantification results are presented in Table 4 and Fig. 5. The mean hemoglobin levels for all the male and female athletes in placebo and Fe-treatment groups were within normal ranges throughout the study. The hemoglobin level of male athletes was higher than that of female athletes. Although, no considerable difference was observed within the placebo group of either male or female athletes after 4 weeks. In contrast, a non-significant increasing trend in hemoglobin concentration was noticed within the Fe-treatment group for both males (Fig. 5a) and female athletes (Fig. 5b). Further, no statistically significant differences could be achieved between placebo and Fe-treatment groups when ANCOVA and repeated measured MANCOVA model to determine the difference in the group-by-time interaction during the study, were applied (see Table 4). A similar pattern of hemoglobin levels was observed in the male and female subjects. Again, the effect sizes associated with iron supplementation on the statistically significant outcomes of the studied biological variables were consistently higher when compared to placebo for both male and female subjects.

**Table 4:** Hemoglobin levels and salivary stress biomarkers response among non-anemic athletes during the iron supplementation over four weeks of training exercise regimen compared to placebo.

Male (Site-1) Variable	Placebo			*Cohen's d*	Fe-treatment			*Cohen's d*	P-Value & F-Value (*Between groups)*	Repeated Measure MANCOVA
	*0W*	*2W*	*4W*		*0W*	*2W*	*4W*		*ANCOVA*	(Group by Time)
**Hemoglobin (g/dL)**	(N = 20)				(N = 21)				0W–2W: P = 0.790; F = 0.07	
*Mean* ± *sem*	14.34 ± 0.35	14.42 ± 0.34^#^	14.46 ± 0.31	2W: 0.04	14.46 ± 0.36	14.21 ± 0.32	14.67 ± 0.30	2W: -0.12	*0W–4W: P* = *0.933; F* = *0.01*	P = 0.721; F = 0.18
*95% CI*	13.65–15.04	13.74–15.09	13.86–15.07	4W: 0.07	13.75–15.16	13.57–14.84	14.09–15.26	4W: 0.13		
**Salivary α-Amylase (KU/L)**	(N = 23)				(N = 26)				0W–2W: P = 0.072; F = 3.40	
*Mean* ± *sem*	12.91 ± 1.99	10.57 ± 1.53	13.48 ± 1.85	2W: -0.24	18.15 ± 3.01	19.27 ± 4.27	19.85 ± 2.48	2W: 0.05	*0W–4W: P* = ***0.047****; F* = *4.16*	P = **0.038**; F = 4.58
*95% CI*	9.00–16.82	7.57–13.56	9.85–17.11	4W: 0.05	12.26–24.05	10.90–27.64	10.98–24.71	4W: 0.10		

Female (Site-2) Variable	Placebo (N = 20)				Fe-treatment (N = 20)				P- Value & F-Value (*Between groups)*	Repeated Measure MANCOVA
	*0W*	*2W*	*4W*		*0W*	*2W*	*4W*		*ANCOVA*	(Group by Time)
**Hemoglobin (g/dL)** {N = 18}										
*Mean* ± *sem*	12.98 ± 0.23	nd	13.10 ± 0.22		12.26 ± 0.25	nd	12.48 ± 0.29		*0W–4W: P* = *0.793; F* = *0.07*	*NA*
*95% CI*	12.53–13.43		12.67–13.54	4W: 0.11	11.77–12.76		11.91–13.06	4W: 0.15		
**Salivary Cortisol (nM)**										
*Mean* ± *sem*	3.00 ± 0.69	nd	4.20 ± 0.45		2.28 ± 0.28	nd	4.52 ± 0.75		*0W–4W: P* = *0.674; F* = *0.18*	*NA*
*95% CI*	1.65–4.35		3.31–5.09	4W: 0.40	1.73–2.82		3.05–5.99	4W: 0.65		
**Salivary IgA (ng/mL)**										
*Mean* ± *sem*	179.9 ± 30.6	nd	105.1 ± 30.7		143.8 ± 11.1	nd	78.2 ± 29.3		*0W–4W: P* = *0.649; F* = *0.21*	*NA*
*95% CI*	120.0–239.8		45.0–165.2	4W: 0.45	122.1–165.6		20.8–135.7	4W: 0.48		

*#: N* = *19; nd: not determined; N* = *Number of subjects; sem* = *Standard error of mean; CI* = *Confidential interval; ANCOVA* = *Analysis of covariance; MANCOVA* = multivariate *analysis of covariance (Repeated measure); Significant P* ≤ *0.05; Trending significant P* ≤ *0.10; NA* = *Not applicable; IgA* = *Immunoglobulin A.; Effect size (Cohen's d)*.

**Fig. 6:**
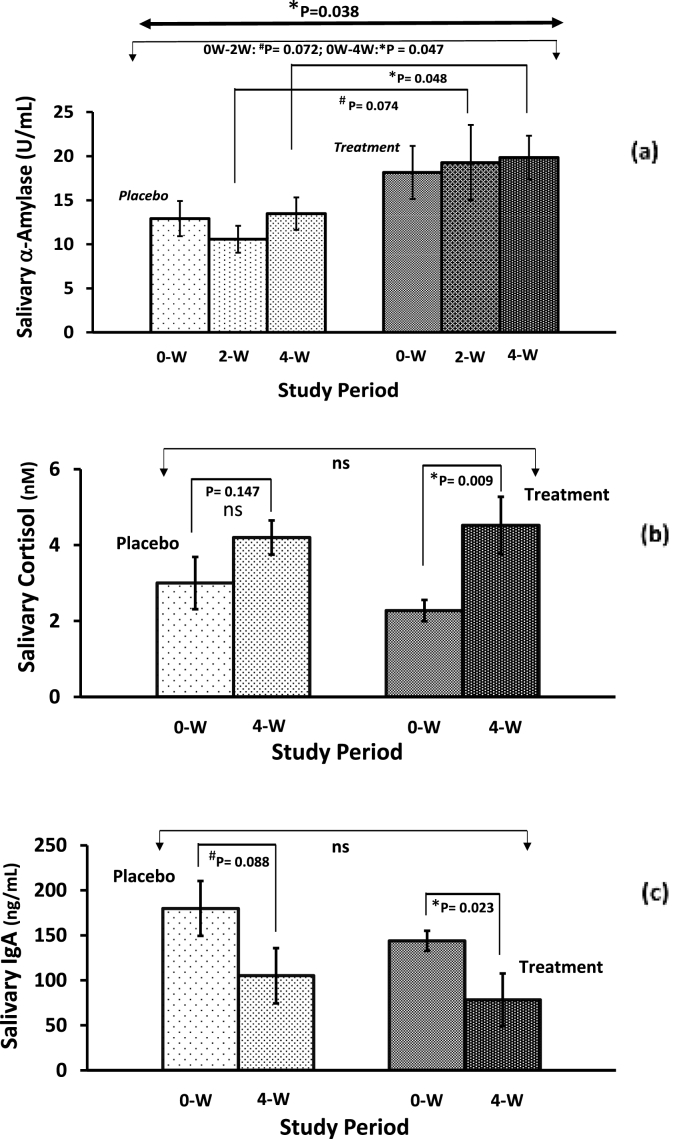
(a) Effect of iron supplementation on differences in salivary α-amylase responsiveness following the four weeks of training exercise regimen among the non-anemic male soccer/futsal player athletes. (b) Saliva cortisol and (c) Saliva immunoglobulin A biomarkers response to low iron supplementation among non-anemic female athletes with sweating training exercise habit. *Keys: As illustrated in*Fig. 1*.*

Fig. 6a depicts the mean of salivary α-amylase, which is considered to be a stress biomarker, in the male athletes over the 4-week study period. When comparing within-groups, initially the salivary α-amylase slightly decreased after 2 weeks in the placebo group, however, at the end of the intervention period, it remained the same as a baseline level. The salivary α-amylase showed a non-significant gradual increase throughout the study period in the Fe-treatment group; there were trending significant differences after 2 weeks (P = 0.074) and significant differences after 4 weeks (P = 0.048) when comparing the placebo and iron supplementation groups. Similarly, trending significant differences (P = 0.072; F = 3.40), and a significant difference (P = 0.047; F = 4.16) were confirmed between placebo and iron supplementation groups after 2 weeks and 4 weeks, respectively, when the ANCOVA model was applied. Further, a statistically significant difference (P = 0.038; F = 4.56) was found between placebo and Fe-treatment groups after 4 weeks when a repeated measured MANCOVA model was applied to determine the difference in the group-by-time interaction during the study period.

A significant within-group difference in the Fe-treatment group (P = 0.009) after 4 weeks was observed for salivary cortisol, while no significant difference (P = 0.147) was noticed in the placebo group (Fig. 6b). There was an increasing trend for salivary cortisol concentrations in both groups, but the levels remained in the normal range (1.4–10.2 nM [59]; throughout the study period. Further, no differences (P = 0.674; F = 0.18) were detected between placebo and Fe-treatment groups after 4-weeks when the ANCOVA model was applied (See Table 4).

The salivary immunoglobulin-A values are presented in Table 4 and reveal a decreasing pattern for both groups. When comparing within groups, a trending decrease (P = 0.088) in the placebo group and a significant decrease (P = 0.023) in the Fe-treatment group were noticed after the 4 weeks intervention period (Fig. 6c). In addition, no statistically significant difference (P = 0.649; F = 0.21) was observed among the mean salivary immunoglobulin-A levels between groups after 4-week intakes when the ANCOVA model was applied.

### 3.5 Salivary biomarkers association with study variables

The correlations between salivary biomarkers (α-amylase, immunoglobulin-A, and cortisol), and fatigue-sweat-related exercise-driven study parameters including POMS-related mood cluster scores were examined using Spearman's rank correlation coefficient to estimate the association between the study variables and depicted in Supplementary Table S4. A significant negative correlation between the degree of sweat and salivary immunoglobulin-A (r = −0.353, P = 0.026) was observed among female athletes at the baseline. The further details are presented in the supplementary information.

To interpret the association of salivary amylase with POMS variables among the male athletes, Spearman's rank correlation coefficient was used to examine correlations, with results presented in Supplementary Table S5. All POMS mood clusters showed weak positive correlations at baseline with salivary amylase. The variables Confusion-Bewilderment (CB) showed a low positive correlation with significant trending (r = 0.246; P = 0.096), while Depression-Dejection (r = 0.313; P = 0.032) and Tension-Anxiety (r = 0.296; P = 0.043) showed the significant low positive correlations at baseline with salivary amylase. However, very low positive correlations were noticed for the aforementioned variables with salivary amylase after the intervention period of 4 weeks of the study. These results support the similar findings reported for the CB, DD, and TA clusters of POMS analysis using the ANCOVA model and repeated measure MANCOVA model to determine the difference in the group by time interaction during the study (see Table 2). On the other hand, a considerably low negative correlation between salivary amylase with Anger-Hostility (AH), Fatigue-Inertia (FI), and Vigor-Activity (VA) was observed after the 4-week intervention period of the study. Total Mood Disturbance (TMD) showed a non-significant very low positive correlation with salivary amylase at baseline as well as after the intervention period of 4 weeks of the study (see Table S5).

## 4 Discussion

Athletes may have an increased prevalence of low iron levels due to a net negative iron balance as a result of exercise. Therefore, it is necessary to precisely monitor the iron status of endurance athletes to maintain the athletes' health and performance because a well-known consequence of iron deficiency is a decline in hemoglobin concentration and fatigue. Also, iron supplementation is not without risk, and the absorption of supplemental iron depends on the preparation used. Some studies revealed no significant changes in physical capacity with iron supplementation among non-anemic athletes. Therefore, we have selected an iron formulation with a known high bioavailability to investigate its effectiveness in non-anemic athletes. In addition, the dosage restriction is also necessary to achieve acceptable compliance because the adverse effects of supplementary iron are dose-dependent. In the present study, athletes were supplemented with a low dose of iron formulation (3.6 mg/day), since high doses of iron supplements are associated with gastric upset. Therefore, a proprietary ferric pyrophosphate salt (SunActive® Fe) was investigated because of its comparatively better iron bioavailability and negligible gastrointestinal problems. Overall, the present study results represent a good argument against the routine use of iron supplementation in endurance athletes.

Although there is limited evidence for an association between iron deficiency and fatigue, some findings revealed iron supplementation may improve fatigue for non-anemic individuals [60]. Physical performance is reported to be reduced in iron deficiency due to inadequate cellular energy production (related to functioning mitochondrial enzymes) as well as burdens on oxygen transport when hemoglobin is reduced among athletes. Other studies have also corroborated the benefits of iron supplements for fatigue reduction [[61], [62], [63], [64], [65], [66], [67]]. Although a low dose of iron supplement (3.6 mg/day) over 4 weeks was not enough to show a higher group-by-time interaction of hemoglobin in the present study; however, it could be associated with exercise driven sweat and subjective fatigue-related variables, and thus represent a reasonable measure of an improvement of subjective fatigue among athletes. Also, a decrease in the degree of sweat among both male and female athletes suggests that maintaining iron status could have controlled the secretory activity of the athlete's sweat gland during physical training. However, the entailed mechanism remains unclear and needs further investigation.

We measured psychological stress levels using the POMS, a sweat-fatigue scale, and VAS questionnaires. A decrease in the burden of exercise, as well as body pain and tired feeling after the exercise during the iron supplementation period, is well corroborated with an increased relief of fatigue and reduction in the degree of sweat in male soccer/futsal players. Almost a similar pattern of results could be noticed except for some inconsistency with tired feelings after the exercise among the female athletes, which possibly be influenced by the common menstrual cycle-related stress indices. While the results support the concept of increased relief in subjective fatigue with low dose iron supplementation, it was difficult to determine clearly the relative burden of this fatigue. The earlier studies [65,66,68] also found no significant difference between iron and placebo intake in producing improvement in fatigue. The current results show the iron status of the athletes was maintained and their fatigue levels dropped or were stable when consuming a low dose (3.6 mg/day) of iron. Although the iron status has been known to be associated with decreased physical activity [69,70] and the performance was linearly and positively correlated with hemoglobin over the entire range normally seen in humans [71], the interpretation of the results of present study remains a challenge to our knowledge base. Furthermore, the contributory role of sweating to iron levels reduction (*i.e.* lowering hemoglobin levels) might be minor related to a single event; however, the prolonged exercising regimens over several training sessions may induce a substantial effect on the iron status. Further, the results suggested an absence of increased erythropoiesis indicating that could be limited to athletes with below normal hemoglobin concentration [72]. Although the hemoglobin values do not necessarily reflect iron status in other compartments of the body, it is suggested that fatigue might occur when the iron status is reduced in the brain tissues. Associated with psychophysiological assessment, the proper analysis of dietary and lifestyle-related parameters especially sleep efficacies scores, will provide the opportunity to identify and link the stress-driven behaviors related to an athlete's performance. Thus, the effect of iron supplementation on exercise stress behavior may be determined by various spatial or temporal factors; these include brain iron levels and their affinity with neurotransmitters as well as iron exposure period and timings.

The biomarkers salivary cortisol, salivary immunoglobulin A, and salivary α-amylase are considered reliable and convenient indicators of the stress response to exercise and change due to the intensity levels of physical exercise and recovery. Salivary cortisol levels can be modulated by the intensity and duration of exercise [73,74]. Salivary cortisol levels reported in the present study are within the reported normal range for healthy adults [59]. We observed that even a low dose of iron supplement helps to maintain normal levels of salivary cortisol which indicates the reduction of exercise-associated stress during the training period since the low levels of cortisol are reported to cause weakness, fatigue, and low blood pressure [41]. Thus, appropriate cortisol levels seem to be necessary to modulate the exercise-induced stress response and optimal recovery from fatigue. Further, cortisol has been proven to exhibit a wide range of deleterious effects on stress responses. Nevertheless, the results of the present study indicate that a significant increase was found in salivary cortisol in response to exercise during the 4-week iron supplementation period. Wherein the changes are most likely either due to circadian variation or possible contemporary psychological stress related to a mild stimulus for hypothalamic pituitary adrenal axis activation.

The measurement of salivary immunoglobulin A is a useful and non-invasive method for measuring stress during physical activity. Its secretion is stimulated by various factors such as stress or physical activity [43], and explained by the activity of the sympathetic nervous system (SNS), which may reduce the amount of saliva or inhibit its secretion depending on the intensity, duration and type of physical activity [33,[75], [76], [77]]. The present study also focused on the salivary immunoglobulin A response to exercise along with dietary iron intake among male athletes (professional soccer/futsal players). The study participants were asked to maintain their exercise pattern and intensity to ensure a consistent level of physiological stress. A significant decrease in salivary immunoglobulin A levels during iron supplementation compared to placebo can be attributed to its synergic effect along with repetitive exercise. Most of the research showed persistent moderate-to-intensive exercise causes a decrease in the salivary immunoglobulin A levels in athletes [78,79]. It is not clear what mechanism causes a synergic effect of iron intake with volume and intensity of regular exercise on the salivary immunoglobulin A level. However, these immunological basis changes in the salivary immunoglobulin A levels in athletes could be explained by the possible changes in cytokine levels such as T-helper 2 (Th2) and IL-5 secretion, which are responsible for salivary immunoglobulin A production. In addition, regulatory T cells (Treg) produce transforming growth factor-B (TGF-B) that could accelerate salivary immunoglobulin A production, and in turn inhibits T-helper 2 cell ontogenesis [[80], [81], [82]]. Further, the significant decrease in salivary immunoglobulin A levels may be attributed to sympathetic nervous system stimulation [83] related to the exercise regimen along with iron supplementation compared to placebo, because simply the moderate-to-intense exercise (soccer-specific intermittent exercise) is reported to unable to evoke sufficient stimulation of the sympathetic nervous system or the hypothalamic-pituitary-adrenal axis to modify salivary immunoglobulin A transcytosis [[84], [85], [86], [87]].

An increased salivary α-amylase activity in athletes is considered to be induced by exercise-driven stress and is also reported to be increased in response to psychological stress among healthy individuals [88,89]. The results of the present study indicate a non-significant steady increase in salivary α-amylase levels during the iron supplementation of 4 weeks while showing a significant difference compared to placebo among the female athletes. It is worth mentioning that the 95% CI ranges of salivary α-amylase reported in Table 4 for placebo and Fe treatment groups during the study periods were always below <30 kU/L, confirming that participants experienced no stress throughout the study period [90]. This suggests a connection between physical stress, iron supplementation, and salivary α-amylase regulation [91].

The present study also compares the POMS scores in the placebo and Fe-treatment groups of male athletes, and the outcomes demonstrate routine exercise might induce changes. Especially, the Depression-Dejection (DD) and Tension-Anxiety (TA) clusters of POMS analysis were significantly altered with iron intake compared to placebo and, thus confirmed the positive impact of iron intake on the total mood disturbance (TMD) among the male athletes. Also, it seems that male athletes in the Fe-treatment group had a greater adaptation to training stress regimes than the athletes from the placebo group since the correlations between salivary α-amylase responses and some of the self-reported cluster's responses in the POMS analysis were significant at baseline and remained positively correlated to the salivary α-amylase. Further, an increase in the salivary α-amylase levels could be linked to the manageable aspect of the physical stress-related situation, wherein an increase in SNS activity may be specifically related to the mobilization effort during a training exercise [92].

Notwithstanding, we did measure the exposure that is most likely to reveal the information on the causes that reflect the alteration in stress behavior among non-anemic endurance athletes, such as lifestyle, food and nutritional habits, sleep quality, and other related form of physical stress and relaxation status. Generally, the changes in body temperature follow a circadian rhythm and are used to assess physiological conditions. A non-significant decrease in the body temperature and ameliorated pulse rate during iron supplementation could be directly associated with improved subjective sleep efficacies scores over four weeks period of study among female athletes. Wherein the sleep parameters such as comfortable sleep, sleep quality, and positive attitude after waking contribute to the improved sleep scores. Such an improvement in the subjective sleep score was further corroborated by significantly improved tension-anxiety (TA) and depression-Dejection (DD) clusters of POMS analysis. Lastly, the fidelity was implemented according to trial protocols and was examined by compliance check. Overall, the fidelity of the present informed intervention study was high at both clinical sites (>99% compliance).

The study provides new insights into stress, mood states, fatigue, and sweating behavior among the non-anemic athletes who consumed a routine low dose of iron. Despite this, the present research has limitations including gender differences and choosing an adequate treatment period. There is a possibility that the study treatment period of four weeks was too short to be fully effective because the repletion of exhausted iron stores is time-consuming even among the non-anemic endurance athletes with no pathological iron loss. Although, we did not find a significant gender effect on any of the measured parameters; however, the other outlined issues are that the effect of physical training on iron balance could be more reliable in males compared to female endurance athletes because the menstrual iron loss that possibly complicates the finding is avoided. The contributory role of sweating to bodily iron loss could be considered negligible among non-anemic athletes; however, the training for prolonged periods may induce a mild cumulative effect on the iron status. Therefore, a routine use of low dose iron supplementation is suggested for endurance athletes to improve the stress, mood states, fatigue, and sweating conditions.

## 5 Conclusion

In summary, the iron supplementation (3.6 mg/day) over 4 weeks in the present double-blind, randomized, placebo-controlled, parallel groups study was shown to be sufficient to prevent a decline in hemoglobin levels and reduce stress (salivary cortisol, salivary immunoglobulin A, and salivary α-amylase) in response to a training regimen among athletes. While 3.6 mg/day of iron supplementation over four weeks did not demonstrate a significant group by time interaction of hemoglobin; however, it was observed that maintained hemoglobin levels were associated with fatigue and sweating behavior of the athletes. The results showed iron supplementation aided the psychological recovery of fatigue and reduced the burden of exercise, rendered relief from tired feelings and significantly improved the total mood disturbance (TMD) among the male athletes. Such a recovery from subjective fatigue could be attributed to the maintenance of iron metabolism and status in the body. Practical implications for the maintenance of iron level in athletes should incorporate dietary modifications along with particular focus on increasing total dietary iron, and improving iron bioavailability by safe commercially available formulations. For instance, Sunactive Fe® consumed in this study is well known for enhanced iron absorption. The present research support the hypothesis of the beneficial effect of dietary iron interventions on the balance of iron in non-anemic endurance athletes. Since the iron deficiency is sport is frequent and affect physical performance, therefore, more systematic studies are warranted to better understand the underlying mechanism of iron-associated fatigue and athletes' stress behavior during exercise sweating. In summary, the take-home message is that even a low dose daily iron supplementation can help maintain and may be useful to improve the overall wellness of non-anemic athletes engaged in endurance exercise, as well as individuals with marked sweat losses due to heavy physical work.

## Author's contributions

Otsuma Women's University, Japan, and Nippon Sport Science University, Japan were responsible for the study design, and data collection, and provided the final study report. All authors contributed equally to this work. MS, MK, TO, and MPK initiated this work. Data were collected by AK, MK, and AA. Interpretation of data, intensive literature research and manuscript preparation were undertaken by MPK, DT, MS, and MK along with required revision. All authors approved the final version of the manuscript after receiving acceptance from their respective research institutes.

## Funding disclosure

The study was performed by Otsuma Women's University, Japan, and Nippon Sport Science University, Japan at their research facilities, and received partial research funding under the contract supported by Taiyo Kagaku Co. Ltd., Japan.

## Declaration of competing interest

The authors declare that they have no known competing financial interests or personal relationships that could have appeared to influence the work reported in this paper.
