# Supplementary material for: Influence of iron supplementation on fatigue, mood states and sweating profiles of healthy non-anemic athletes during a training exercise: A double-blind, randomized, placebo-controlled, parallel-group study
Source: Contemp Clin Trials Commun. 2023 Feb 3;32:101084. doi: 10.1016/j.conctc.2023.101084 (PMC9932653; doi:10.1016/j.conctc.2023.101084)
Supplement: Multimedia component 1 [file mmc1.docx]

*Supplementary Information* (CONCTC-D-22-00123; Kapoor et al.)

**Influence of iron supplementation on fatigue, mood states and sweating profiles of healthy non-anemic athletes during a training exercise: a double-blind, randomized, placebo-controlled, parallel-group study**

Mahendra P. Kapoor^1*^, Masaaki Sugita ^2^, Mikiko Kawaguchi ^3^, Derek Timm ^4^, Aki Kawamura^2^, Aya Abe ^1^, Tsutomu Okubo^1^

*^1^ Taiyo Kagaku Co., Ltd., Research & Development, Nutrition Division, 1-3 Takaramachi, Yokkaichi, Mie 510-0844, Japan.*

*^2^ Nippon Sport Science University, Faculty of Sport Science, 7-1-1 Fukusawa, Setagaya-Ku, Tokyo 158 8508, Japan.*

*^3^ Otsuma Women's University, Faculty of Home Economics, Department of Food Science, 12 Sanbancho, Chiyoda, Tokyo 102-8357, Japan.*

*^4^Taiyo International Inc. 5960 Golden Hills Dr., Minneapolis, MN 55416, USA.*

E-mail address: [mkapoor@taiyokagaku.co.jp](mailto:mkapoor@taiyokagaku.co.jp) (M. P. Kapoor)

*Submitted to:*

*Journal:* *Contemporary Clinical Trials Communications (Kapoor et al.)*

Revised: CONCTC-D-22-00123; R1-Kapoor et al.

25^th^ January 2023

**Table S1.** The daily dietary consumption among non-anemic athletes of both placebo and Fe-treatment groups at either clinical site monitored prior to the study, and the breakdown into weekly dietary intake subgroups is displayed.

| **Description** | ***Site-1 (Male athletes)*** | | ***Site-2 (Female athletes)*** | |
| --- | --- | --- | --- | --- |
|  | **Placebo** | **Fe-treatment** | **Placebo** | **Fe-treatment** |
|  | *Score (per day)** | | | |
| Breakfast | 1.6 ± 0.7 | 1.7 ± 0.7 | 1.2 ± 0.3 | 1.1 ± 0.3 |
| Lunch | 2.1 ± 0.2 | 2.1 ± 0.2 | 1.5 ± 0.2 | 1.4 ± 0.2 |
| Dinner | 1.8 ± 0.4 | 2.0 ± 0.6 | 1.4 ± 0.3 | 1.3 ± 0.2 |
|  | *Score (per week)** | | | |
| Meat products (Beef, Pork, Chicken etc.) | 5.4 ± 1.7 | 5.5 ± 1.9 | 9.0 ± 2.2 | 9.2 ± 1.3 |
| Fish and sea food products | 2.4 ± 1.9 | 2.5 ± 1.2 | 5.8 ± 2.0 | 6.2 ± 1.9 |
| Egg products | 4.0 ± 2.4 | 4.1 ± 2.3 | 4.6 ± 1.3 | 4.5 ± 1.1 |
| Beans products | 5.5 ± 1.9 | 3.9 ± 2.0 | 4.3 ± 1.4 | 4.5 ± 1.3 |
| Milk and dairy products | 5.1 ± 2.7 | 4.9 ± 2.9 | 7.7 ± 2.2 | 6.9 ± 2.5 |
| Vegetable products (Green and others) | 5.7 ± 1.9 | 4.8 ± 2.1 | 6.1 ± 3.1 | 6.3 ± 2.6 |
| Fruits and processed products | 2.3 ± 2.0 | 3.2 ± 2.3 | 7.1 ± 2.6 | 6.4 ± 1.8 |
| *Score points scale:* ***0****- Do not eat;* ***1****-Eat a little;* ***2****- Moderate eating ;* ***3****- Eat too much* | | | | |
| *Score scale:* ***0****- Do not eat;* ***1****- Once a week;* ***2****- Twice a week ;* ***3.5*** *- 3 or 4 times per week;* ***5.5*** *- 5 or 6 times per week;* ***7*** *-Eat every day* | | | | |
| *No significant difference between groups; Athletes maintained the dietary intake over 4 weeks of study duration; Significant P ≤ 0.05; | | | | |

**Table S2.** The detailed breakdown into weekly exercise regimen subgroups of the female athletes is displayed.

| **Description of Exercises** | ***Site-2 (Female athletes)*** | |
| --- | --- | --- |
|  | **Placebo** | **Fe-treatment** |
|  | *min/day* | *min/day* |
| Yoga /Hot yoga exercises | 23.3 ± 31.3 | 23.7 ± 26.5 |
| Resistance movements | 3.6 ± 10.9 | 3.2 ± 8.5 |
| Martial arts exercises | 2.1 ± 6.7 | 2.2 ± 5.3 |
| Dance exercises | 1.1 ± 4.7 | 1.3 ± 5.6 |
| Underwater exercises | 1.1 ± 5.0 | 1.1 ± 5.0 |
| Walking | 0.7 ± 3.1 | 0.6 ± 2.7 |
| Running | 0.3 ± 1.2 | 0.0 ±0.1 |
| Other miscellaneous exercises | 0.9 ± 3.7 | 1.4 ± 6.2 |
| *Total exercise duration (min/day)* | *33.0 ± 66.7* | *33.6 ± 59.9* |
| **No significant difference between groups;* | | |
| *Athletes maintained the exercise regimen over 4 weeks of study duration; Significant P ≤ 0.05;* | | |

**Table S3.** The effect of iron supplementation on sleep quality and anthropometric measures following the four-week exercise regimen among non-anemic female athletes.

| **Variable (scores)** | **Site-2; Female** | | | | | | | |
| --- | --- | --- | --- | --- | --- | --- | --- | --- |
|  | **Placebo (N= 21)^#^** | | | **Fe-treatment (N= 21)^#^** | | | **P- Value & F-Value (*Between groups)*** | |
|  | *0W* | *2W* | *4W* | *0W* | *2W* | *4W* | *ANCOVA* | MANCOVA  Repeated measure  (Group by Time) |
| **Sleepiness** |  |  |  |  |  |  | *0W-2W :P= 0.479; F = 0.51* | P= 0.691; F = 0.16 |
| *Mean± sem* | 71.32 ± 5.45 | 73.82 ± 4.65 | 66.54 ± 4.91 | 71.85 ± 4.86 | 69.77 ± 4.79 | 75.86 ± 4.06 | *0W-4W :P= 0.152; F = 2.14* |  |
| *95% CI* | 60.63 - 82.00 | 64.71 -82.93 | 56.93 -76.16 | 62.33 - 81.38 | 60.39 - 81.38 | 67.89 -83.82 |  |  |
| **Sleep quality** |  |  |  |  |  |  | *0W-2W : P= 0.730; F= 0.12* | P= 1.0; F = 0 |
| *Mean± sem* | 59.84 ± 6.10 | 62.12 ± 4.53 | 62.77 ± 4.90 | 58.56 ± 4.69 | 64.48 ± 5.01 | 67.00 ± 4.76 | *0W-4W :P= 0.484; F = 0.50* |  |
| *95% CI* | 47.88 - 71.79 | 53.24 - 70.99 | 53.16 - 72.37 | 49.37 - 67.74 | 54.66 - 74. 30 | 57.68 - 76.32 |  |  |
| **Feeling upon awakening** |  |  |  |  |  |  | *0W-2W :P= 0.649; F= 0.21* | P= 0.287; F = 1.3 |
| *Mean± sem* | 59.94 ± 6.00 | 66.73 ± 4.42 | 59.69 ± 4.76 | 48.31 ± 5.83 | 60.34 ± 4.41 | 59.10 ± 5.08 | *0W-4W :P= 0.455; F = 0.57* |  |
| *95% CI* | 48.18 - 71.70 | 58.07 - 75.39 | 50.37 - 69.02 | 36.88 - 59.74 | 51.70 - 68.99 | 49.15 - 69.04 |  |  |
| **Total sleep efficacy score** |  |  |  |  |  |  | *0W-2W :P= 0.842; F= 0.04* | P= 0.831; F = 0.04 |
| *Mean± sem* | 63.70 ± 4.64 | 67.56 ± 3.62 | 63.00 ± 4.13 | 59.76 ± 3.49 | 64.67 ± 3.95 | 67.32 ± 3.77 | *0W-4W :P= 0.691; F = 0.16* |  |
| *95% CI* | 54.61 - 72.78 | 60.45 - 74.66 | 54.91 - 71.09 | 52.92 - 66.59 | 56.93 - 72. 41 | 59.93 - 74.70 |  |  |
| **Pulse rate (bpm)** |  |  |  |  |  |  |  |  |
| *Mean± sem* | 69 ± 2 | nd | 73 ± 2 | 70 ± 2 | nd | 75 ± 1 | *0W-4W :P= 0.808; F = 0.06* | *NA* |
| *95% CI* | 65 - 73 |  | 70 - 78 | 65 - 74 |  | 72 - 77 |  |  |
| **Body Temperature (°C)** |  |  |  |  |  |  |  |  |
| *Mean± sem* | 36.24 ± 0.14 | nd | 36.16 ± 0.13 | 36.39 ± 0.10 | nd | 36.19 ± 0.14 | *0W-4W :P= 1; F = 0* | *NA* |
| *95% CI* | 35.97 - 36.51 |  | 35.91 - 36.41 | 36.19 - 36.59 |  | 35.91- 36.46 |  |  |
| **Body weight (Kg)** |  |  |  |  |  |  |  |  |
| *Mean± sem* | 55.1 ± 2.1 | nd | 55.1 ± 2.2 | 56.2 ± 2.3 | nd | 55.4 ± 2.3 | *0W-4W :P=* ***0.006*****; F = 2.53* | *NA* |
| *95% CI* | 51.0 - 59.2 |  | 50.9 - 59.4 | 51.6 - 60.8 |  | 50.8 -59.9 |  |  |
| *N= Number of subjects; sem= Standard error of mean; CI = Confidential interval; ANCOVA= Analysis of covariance; MANCOVA= Multivariate analysis of covariance (Repeated measure); Significant P* ≤ *0.05; Trending significant P* ≤ *0.10; NA= Not applicable; nd: not determined; #: Non- significant (P > 0.05) within-group (ANOVA); bpm: beats per minute* | | | | | | | | |

**Table S4.** Correlation coefficients between the salivary stress biomarkers and exercise-driven variables related to fatigue, sweating and body physical conditions reveal their associations over four weeks of exercise among non-anemic athletes.

| **Variables** | **Site-1; Male** | | **Site-2; Female** | | | |
| --- | --- | --- | --- | --- | --- | --- |
|  | **Salivary α- Amylase** | | **Salivary IgA** | | **Salivary Cortisol** | |
|  | *0W* | *4W* | *0W* | *4W* | *0W* | *4W* |
|  | *r- value (P- value)* | *r -value (P- value)* | *r- value (P- value)* | *r -value (P- value)* | *r- value (P- value)* | *r -value (P- value)* |
| **Burden of Exercise** | 0.103 (0.497) | 0.117 (0.438) | ̶ 0.060 (0.713) | 0.051 (0.722) | ̶ 0.018(0.914) | 0.097 (0.550) |
| **Body Pain: Post Exercise** | 0.026 (0.866) | ̶ 0.036 (0.812) | ̶ 0.165 (0.309) | ̶ 0.086 (0.598) | 0.124 (0.447) | ̶ 0.016 (0.921) |
| **Tiered Feeling: Post Exercise** | ̶ 0.172 (0.251) | 0.023 (0.882) | ̶ 0.258 (0.108) | ̶ 0.069 (0.673) | 0.133 (0.415) | 0.001 (0.995) |
| **Relief from Fatigue** | ̶ 0.184 (0.227) | ̶ 0.077 (0.609) | 0.001 (0.996) | 0.003 (0.988) | 0.190 (0.239) | 0.026 (0.871) |
| **Degree of Sweat** | 0.055 (0.717) | 0.101 (0.505) | ̶ 0.353 (**0.026***) | 0.040 (0.801) | ̶ 0.012 (0.940) | 0.058 (0.721) |
| **Significant P* ≤ *0.05; ^#^Trending significant P* ≤ *0.10; Spearman rank correlation; IgA: Immunoglobulin A* | | | | | | |

While a non-significant very low negative correlation for salivary immunoglobulin-A with the burden of the exercise was noticed. However, after the 4 weeks intervention period, the very low positive correlations observed for both variables support the decreasing tendency of the degree of sweat and burden of exercise results reported for female athletes (*see Table 3*). Similarly, the non-significant very low negative correlations at baseline observed for salivary cortisol with the degree of sweat and burden of exercise revealed a very low positive correlation after the intervention period of 4 weeks and further confirmed the decreasing tendency of the degree of sweat and burden of exercise results reported for female athletes. Other variables such as body pain after exercise, relief from fatigue, and tired feeling after exercise showed similar correlations for salivary immunoglobulin-A, and salivary cortisol at baseline as well as after the intervention period of 4 weeks of the study. Further, among the male athletes, a non-significant very low negative correlation noticed at baseline for salivary amylase with tired feeling after the exercise, also revealed a very low positive correlation after the intervention period of 4 weeks, evidently supporting the results reported for a significant decrease in tendency of feeling tired after the exercise among male athletes (*see Table 3*). On the other hand, while the variable body pain after exercise showed non-significant a very low positive correlation for salivary amylase at the baseline, revealed a very low negative correlation after the 4 weeks intervention period without any significant difference. Whereas other variables showed no change in their correlations for salivary amylase at baseline as well as after the intervention period of 4 weeks of the study.

**Table S5.** Correlation coefficients between the salivary α-amylase and profile of mood states (POMS) clusters reveals their association over four weeks of exercise among non-anemic male athletes.

| **POMS Variables** | **Site-1; Male** | |
| --- | --- | --- |
|  | **Salivary α- Amylase** | |
|  | *0W* | *4W* |
|  | *r- value (P- value)* | *r -value (P- value)* |
| **Anger-Hostility (AH)** | 0.092 (0.537) | ̶ 0.009 (0.951) |
| **Confusion-Bewilderment (CB)** | 0.246 (**0.096^#^**) | 0.033 (0.823) |
| **Depression-Dejection (DD)** | 0.313 (**0.032***) | 0.002 (0.988) |
| **Fatigue-Inertia (FI)** | 0.145 (0.332) | ̶ 0.003 (0.986) |
| **Tension-Anxiety (TA)** | 0.296 (**0.043***) | 0.062 (0.680) |
| **Vigor-Activity (VA)** | 0.144 (0.334) | ̶ 0.011 (0.942) |
| **Total Mood Disturbance (TMD)** | 0.185 (0.213) | 0.049 (0.745) |
| **Friendliness (F)** | 0.236 ( 0.111) | 0.015 (0.919) |
| **Significant P* ≤ *0.05; ^#^Trending significant P* ≤ *0.10; Spearman rank correlation* | | |
